# Water Consumption in European Children: Associations with Intake of Fruit Juices, Soft Drinks and Related Parenting Practices

**DOI:** 10.3390/ijerph14060583

**Published:** 2017-05-31

**Authors:** Krystallia Mantziki, Carry M. Renders, Jaap C. Seidell

**Affiliations:** Department of Health Sciences, Faculty of Earth & Life Sciences, Vrije Universiteit Amsterdam, Amsterdam Public Health Research Institute, 1081 HV Amsterdam, The Netherlands; carry.renders@vu.nl (C.M.R.); j.c.seidell@vu.nl (J.C.S.)

**Keywords:** soft drinks, fruit juices, water intake, parenting practices, European communities

## Abstract

*Background*: High intake of fruit juices and soft drinks contributes to excessive weight gain and obesity in children. Furthermore, parenting practices play an important role in the development of children’s dietary habits. The way parents play this role in the development of their children’s choices of beverages is still unclear. *Objectives*: To study the associations: (1) of both fruit juices and soft drinks consumption with water consumption of children and (2) The associations between parenting practices towards fruit juices and soft drinks and water consumption of children. *Design*: Cross-sectional data from 6 to 8 year old children from seven European communities (*n* = 1187) were collected. Associations among fruit juices, soft drinks, the respective parenting practices and the child’s water consumption were assessed by parental questionnaires. *Results*: The consumption of water was inversely associated with that of soft drinks but not with the consumption of fruit juices. The child’s water intake was favorably influenced when stricter parenting practices towards soft drinks were adopted (e.g., less parental allowance, low home availability and high parental self-efficacy in managing intake). There was less influence observed of parenting practices towards fruit juices. Fruit juices were consumed more often than soft drinks. *Conclusions*: Low consumption of soft drinks—and not of fruit juices—was associated with high water consumption in children in the current study. Moreover, parenting practices towards both fruit juices and soft drinks were associated with the water intake of the children, irrespective of their socio-economic status.

## 1. Introduction

Over the past three decades, the global prevalence of overweight and obesity has reached epidemic levels in children [1], while the impacts on health and the relevant economic burden affect individuals as well as the society as a whole [2]. In 2008, about 25% of the children (6–9 years old) in the European region were overweight or obese [3], and this percentage increased to 33% in 2010 [4]. It was characterized as a “worrying increase” in the European Action Plan on Childhood Obesity 2014–2020 [4]. 

It is now generally accepted that a high intake of free sugars contributes to excess weight gain, particularly regarding those sugars contained in beverages [5,6,7,8,9,10,11,12,13]. Currently, limiting the intake of free sugars to a maximum of 10% (and to 5% as conditional recommendation) of the total energy intake is highly recommended [12]. Sugary drinks, including—among others—soft drinks, energy drinks, fruit juices (100% juices included), are high in free sugar content [5,6,7,9,10,11,13]. With the exception of 100% fruit juices, which may contain vitamins and minerals, sugary drinks provide “empty” calories with no nutritional benefit and, thus, lead to low satiety response [7,11,14]. Consequently, high consumption of such drinks is not compensated by a decreased caloric intake from other foods or beverages, hence resulting in increased energy intake and weight gain [11,14]. Moreover, high intake of sugary beverages has been also associated with tooth decay, hyperactivity and mental health problems in children and adolescents [15,16,17,18,19]. Therefore, soft drinks are increasingly perceived by parents as unhealthy, unlike fruit drinks and juices, which are considered as the ”healthy” choice or a healthier alternative to soft drinks [5,7,19], However the sugar content of fruit juices is very similar to that of sugar sweetened soft drinks.

Children’s consumption of fruit juices and soft drinks in western countries has increased considerably during the past decades [6,7,10,15,17,19], while an increase in the intake of sugary beverages seems to occur during the period from childhood to adolescence [9]. For that reason, several intervention studies have examined the effects of the replacement of sugary beverages’ consumption by the consumption of water, in which a reduction in total calories [9,20,21], positive behavioural changes and weight loss have been demonstrated [20]. Nevertheless, discouraging children from drinking sugary drinks and switching to water is challenging, considering the many physical and social environmental factors that may influence their choices. It is well understood that parents play a key role in shaping the development of children’s behaviours [6,10,16,20,21,22], as well as that parents appear to highly impact the development of early childhood overweight [23]. Consequently, as parents are role models for their children, it is of crucial importance to influence parenting practices, beliefs and attitudes towards sugary drinks’ consumption, as well as parental behaviours to achieve behavioural change in children.

Although numerous studies have assessed the association of parental rules and practices regarding sugary fruit juices and/or soft drinks [10,16,19], little is known about the association between these practices and water consumption. As parents play an important role in the development of children’s dietary habits, the aim of this study was to find indications for action for interventions aimed at improving parenting practices towards fruit juices and soft drinks and especially the water consumption of the child. Therefore, the objectives of the current study were: (1) to assess the associations of both fruit juices and soft drinks consumption with water consumption of children and (2) to assess the associations between parenting practices towards fruit juices and soft drinks and the water consumption of children. This research is based on self-reported data derived from a cross-sectional study.

## 2. Materials and Methods

This study is part of the two-year prospective evaluation study of the Epode for the Promotion of Health Equity (EPHE) project, the methodology and aims of which are described elsewhere [24].

### 2.1. Sample and Recruitment

Seven community-based programmes, a part of the Epode International Network, which implemented the EPODE methodology, participated in the EPHE project: VIASANO (Belgium), Ensemble, Prévenons l'Obésité Des Enfants (EPODE) (France), PAIDEIATROFI (Greece), Maia Healthy Menu (Portugal), Si Eu Trăiesc Sănătos! (SETS) (Romania), Jorgeren Op Gezond Gewicht (JOGG) (The Netherlands), HEALTHY KIDS (Bulgaria); the latter programme is part of Nestle’s Healthy Kids programme and implemented a methodology similar to EPODE’s. Every programme is represented by one or two communities. We aimed at recruiting a minimum of 150 families with children in the age group of 6–8 years in every selected community, with a similar variation regarding age and ethnicity per site. The participants were recruited from several schools and the permission to carry out the study in schools was acquired from the local community and/or school authorities, where necessary. More information about the sampling and recruitment processes as well as the response rates of the baseline measurements are described elsewhere [24,25].

### 2.2. Data Collection

The EPHE parental questionnaire [24], a self-administered questionnaire based on relevant, validated questionnaires addressed in European populations [26,27,28] was used for the collection of data. Additional items regarding water consumption and related determinants were constructed, since, to our knowledge, no such validated items existed. The rationale and the development of the questionnaire are described in detail elsewhere [24]. The questionnaires, including an informed consent, were distributed to the children at school and delivered to their parents, before and immediately after the intervention period. After a specified period of one to two weeks, the completed questionnaires were collected and only the ones including a signed statement of informed consent were taken into consideration. In order to assure the confidentiality of the data, a process that ensured the anonymity of the participant families was took place [24].

### 2.3. Measures

#### 2.3.1. Beverage Consumption

We defined as beverage consumption the intake of water, fruit juices and soft drinks. Water intake included water from the tap or from bottles (artesian well water, spring water, mineral water and sparkling water). Fruit juices included those made from both concentrated and 100% freshly blended fruit. As soft drinks were defined: the carbonated drinks, fruit squash/cordials and sport and energy drinks. To assess the consumption of fruit juices and soft drinks, two items from a validated parental questionnaire [28] were used, measuring the weekly frequency on a 6-point Likert scale: (1) Never; (2) Less than once a day; (3) 2–4 times a week; (4) 5–6 times a week; (5) Every day, once a day; (6) Every day, more than once a day [24,25]. To assess the water consumption, we constructed an item that measured its daily frequency on a 6-point Likert scale: (1) Never; (2) Less than once a day; (3) Once a day; (4) 2–4 times a day; (5) 5–6 times a day; (6) More than 6 times a day.

#### 2.3.2. Parenting Practices Regarding Sugary Beverages

The parenting practices measured were: (i) paying attention/monitoring, (ii) Parental allowance, (iii) Negotiating, (iv) Communicating health beliefs regarding soft drinks, (v) avoiding negative modelling, (vi) Parental self-efficacy to manage child’s intake, (vii) Rewarding/comforting practice, (viii) Parent(s) drinking beverage together with the child and (ix) Home availability. Most parenting practices were assessed by one item using a 5 point Likert-scale: (0) Never; (1) Not often; (2) Sometimes; (3) Often; (4) Always. Parental allowance and Communicating health beliefs regarding soft drinks were measured by two items, using the same 5-point scale. The item labeled as Parent drinking beverage together with the child was assessed by using an 7-point Likert-scale: (1) Never; (2) Less than once a week; (3) once a week; (4) 2–4 times a week; (5) 5–6 times a week; (6) Every day, once a day; (7) Every day, more than once a day; (8) Every day more than once a day. More details are described elsewhere [24,25] and are presented in the table of Appendix A.

#### 2.3.3. Socio-Demographic Measures

Socio-demographic characteristics were measured in categorical scales: (a) age of the respondent: (1) <20, (2) 20–24, (3) 25–30, (4) 31–35, (5) 36–40, (6) >41; (b) age of the child: (1) 6 years, (2) 7 years, (3) 8 years, (4) 9 years and above; (c) parental education level in years: (1) <6, (2) 6–8, (3) 9–11, (4) 12–14, (5) 15–17, (6) >17 [27]. The maternal educational level was used as an indicator for the socio-economic status (low-high).

### 2.4. Statistical Analysis

Socio-demographic characteristics were described in terms of percentages (gender of child, age of mother, education level of mother) and means (SD (age of the child)), as presented in Table 1. For each country’s sample, the median of the educational level was used as the cut-off point for defining a low or high educational level of the mother. The available data from the total baseline EPHE sample (May–June 2013) was used, in order to assess the associations between: (a) fruit juices consumption, (b) soft drinks consumption, (c) parenting practices towards fruit juices, (d) parenting practices towards soft drinks and children’s water consumption. For the purposes of this study, binary logistic regression models were adopted to calculate odds ratios and 95 percent confidence intervals (OR and 95% CI respectively). All the measured variables were categorical. As was the dependent variable, the frequency of water consumption was dichotomized on the basis of the median (Md = 5), due to skewed distribution, into low (<5–6 times a day) and high (>5–6 times a day or more) consumption. The independent variables were recoded into three and not in two categories to avoid r loss of information. Specifically, the frequency of consumption of fruit juices and soft drinks was recorded as: (1) low frequency (<once a week), (2) Moderate frequency (2–4 days a week), (3) High frequency (>5–6 days a week). Furthermore, the parenting practices were re-categorised into three categories: (1) low frequency (not often-never), (2) Moderate frequency (sometimes), (3) High frequency (often-always). As shown in descriptive analyses published elsewhere [25], beverages consumption and parenting practices differed from country to country; and thus, tests regarding potential confounding and effect modification were carried out. The educational level of the mother was also assessed with respect to potential confounding and effect modification.

## 3. Results

### 3.1. Socio-Demographic and Descriptive Characteristics

A total of 1266 children and their families were involved in the baseline survey. Due to missing data in the variable “educational level of mother”, finally 1187 subjects were included in the analysis (Table 1). Descriptive analysis showed that the highest frequency of water consumption occurred in the Bulgarian sample, whereas the samples from Belgium and The Netherlands yielded the lowest results (Table 2).

The highest frequency of fruit juices consumption was detected in the samples from Belgium and France, and the lowest in the Dutch sample (Table 2). Moreover, soft drinks were consumed in the highest frequencies by the Belgian participants and in the lowest by the Greek participants (Table 2).

### 3.2. Associations of Fruit Juice and Soft Drinks Intake with Water Consumption

**Figure 1:**
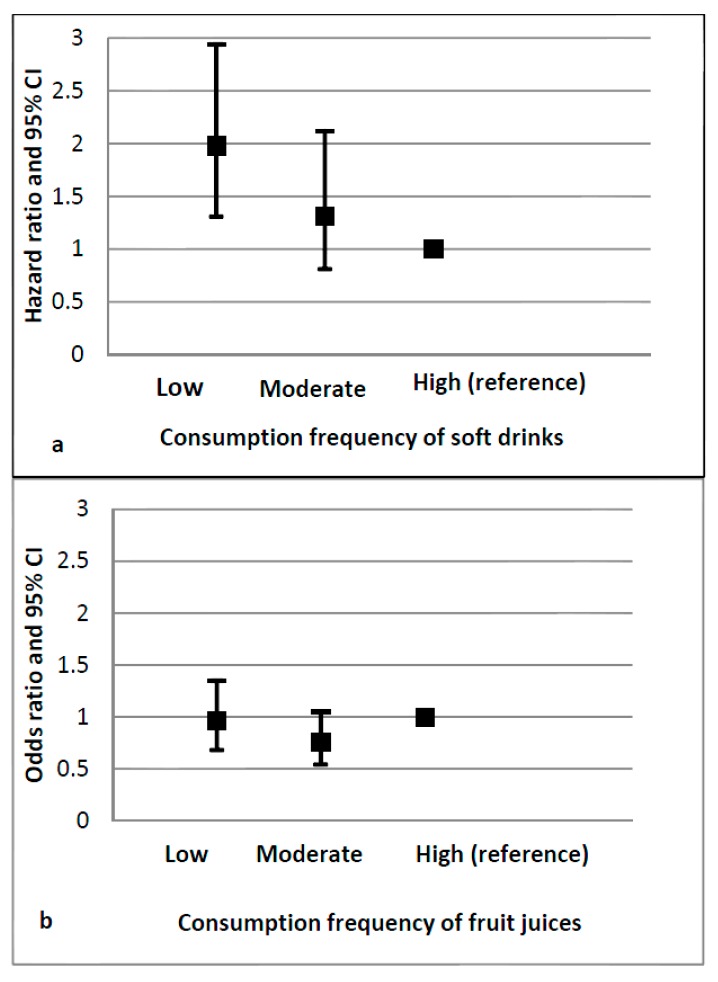
Odds ratios (95% CI) for the frequency of soft drinks (**a**) and fruit juices (**b**) consumption in relation to water consumption (high vs. low), adjusted for the country and educational level of the mother.

Figure 1 illustrates that the lower the frequency of sugary beverages consumption, the higher the odds for a child to consume water in high frequency. However, only the low frequency of soft drinks intake—and not the moderate one—was significantly associated with high water consumption frequency (Figure 1a). The frequency of fruit juices consumption was not associated with the one regarding water (Figure 1b). The education level of the mother and the country did not significantly modify the aforementioned associations, but they proved to be confounders.

### 3.3. Associations between Parenting Practices towards Sugary Beverages and Children’s Water Consumption

In Table 3 and Table 4, the associations between frequency of water intake and parenting practices regarding fruit juices and soft drinks, are presented. The associations were adjusted for country, because this proved to be a confounder. In general there was no effect modification by mothers’ education level, therefore results are presented for the total group.

As shown in the tables when parents monitored their child’s fruit juices/soft drinks consumption in moderate frequency, their children were less likely to consume water in high frequency (i.e., five or more times) compared to the children of parents who monitored their fruit juices/soft drinks intake in high frequency. In addition, children of parents with high self-efficacy in managing the child’s intake of fruit juices and soft drinks were 1.83 (95% CI: 1.04–3.20) and 2.44 (95% CI: 1.26–4.73) times, respectively, more likely to consume water five or more times a day compared to the ones having parents with low self-efficacy. Another finding was that when parents told *sometimes* their children that fruit juices make him/her fat, their children were less likely (OR 0.63; 95% CI 0.41–0.98) to drink water in high frequency compared to the cases when parents communicated the belief *often/always*. Moreover, the low and moderate parental allowance to drink soft drinks was associated with a higher possibility for the child to drink water in higher frequency than the high parental allowance.

When children were communicated a health belief in low frequency, they were less likely to consume water five or more times a day compared to the children whose parents had communicated the health belief to them in high frequency. Also, parents who reported that they drink soft drinks with their children *sometimes* were associated with lower odds (0.55 (95% CI: 0.32–0.96)) for the child to consume water in high frequency compared to parents who reported that they drink soft drinks with the child *often* and/or *always*. Finally, it was found that when the home availability of soft drinks was low, the odds for the children to consume water in high frequency was higher.

## 4. Discussion

This study demonstrated that children who exhibited a low (<once a week) frequency of soft drinks consumption as well as the ones with a moderate one (1–4 times a week) were more likely to drink water in high frequency (>5–6 times a day). Moreover, fruit juices consumption was not associated to water consumption. In addition, the children’s frequency of water intake was strongly influenced by parenting practices towards soft drinks , such as monitoring of child’s intake and by communicating their health beliefs as well as, remarkably, by parental allowance, parental efficacy and home availability. The associations regarding the parenting practices towards fruit juices and water consumption were weaker and significant only with respect to monitoring the child’s intake and the parental efficacy to retain rules, as well as communicating the health belief. Although the parental education level has been associated with sugary beverages in several studies [16,19,29,30], it was not associated with water frequency in our study. Additionally, the associations between parenting practices and water consumption did not differ a lot per education group. Finally, in all EPHE samples, fruit juices were found to be consumed more often than soft drinks, as shown in other studies as well [15,31].

To the best of our knowledge, this is the first study that assessed the influence of parenting practices related to sugary beverages on the water consumption of their children, whereas a growing mass of evidence demonstrates that parenting practices and/or rules influence children’s consumption of sugary beverages. Van Grieken et al. showed that (a) home availability, (b) difficulty to limit the child’s consumption, (c) discouraging sugary beverages consumption by the child (d) not allowing the child to consume sugary beverages and (e) the habit to limit child’s consumption were primary parental practices associated with sugary beverages consumption of children [30]. Relevant studies examining the child’s micro-environment, yielded similar findings [10,16,19,29], although little is known about the impact of parental monitoring on child’s drinking behaviour. The influence of parental monitoring on child’s weight status and behaviour has been shown in several studies [32,33,34,35], but the evidence is mixed [36]. The relatively weak influence of parenting practices towards fruit juices on child’s water consumption can possibly be explained by the fact that a lot of parents perceive fruit juices and/or drinks as a healthy choice in contrast to soft drinks that are perceived as an unhealthy choice. Parents are often not aware of the similar sugar content of both drinks [5,7]. Therefore they may less use parenting practices to stimulate the healthy choice of drinking water when drinking behaviour of fruit juices and/or drinks is involved than when drinking behaviour of soft drinks is involved. 

### 4.1. Methodological Considerations

This study used data obtained from communities of seven different countries in the European region in order to explore trends in beverage consumption and their association with parenting. The cross-sectional sample, a result of high participation rates (65–97.5%) [25], enabled us to explore the influence of parenting practices related to common sugary beverages on the daily frequency of children’s water intake. By assessing fruit juices and soft drinks separately and distinguishing the parenting practices into the ones that influence fruit juices and the ones that impact soft drinks consumption, it was made possible to detect the differing behavioural, consumption and possibly perception patterns of parents and their children regarding the two distinguished categories of sugary beverages. A strength is that, in addition to weekly consumption of fruit juices and soft drinks, we assessed also the daily consumption of water, which is rarely measured. Furthermore, sugary beverages consumption and parenting practices were assessed through validated question items, which yielded moderate to good intraclass correlation coefficients [37]. Nevertheless, the consumption of beverages was self-reported and the same applies to the parenting practices. Therefore, socially desirable answers and recall bias cannot be ruled out. Finally, this is an observational study and, as a result, conclusions about causality cannot be drawn.

### 4.2. Implications to Public Health Research

This research showed that it is important to consider the influence of parenting practices towards sugary beverages, especially that of soft drinks, when it comes to lifestyle patterns of the child, such as water consumption. Therefore, it is worthwhile to have a more detailed assessment of the causal relationship between these factors. In addition, a quantitative assessment of water, fruit juice and soft drinks intake by means of continuous variables would allow application of more elaborative statistical methods and thus may lead to more insights regarding the independent contributions of various predictors.

### 4.3. Implications to Public Health Practice

Interventions aiming to promote healthy lifestyles in children often include attempts to reduce the intake of sugary beverages. Such interventions may benefit from addressing parenting practices and behaviours. Empowering parents to shape a healthy family environment for the child as well as promote water consumption instead of sugary beverages is highly recommended. Finally, it seems important to increase awareness, especially among parents, about the sugar content in all kinds of fruit juices. This may support them to use parenting practices to stimulate their child to make healthy choices for drinks that contain low or no sugar not only when soft drinks but also when fruit juices are involved.

## 5. Conclusions

The current study showed that low consumption of soft drinks—and not of fruit juices—is associated with high water consumption in children. Furthermore, parenting practices towards both fruit juices and soft drinks are associated with the water intake of the children, irrespective of their socio-economic status. Specifically discouraging the consumption of soft drinks may increase water consumption. Moreover, the “healthy” perception of fruit juices/drinks might encourage children to consume these instead of water. Therefore, parenting practices and behaviours seem to be important targets to be addressed in an effort to shape healthy dietary behaviours in children, considering also that the parents are the ones who determine the family environment with respect to food.

## Figures and Tables

**Table 1:** Socio-demographic characteristics of the EPHE population.

Programme, *Country*	Gender		Age Child (Years)	Age of Mother ^a^		Educational Level Mother		Total *n* ^b^
	Boys (%)	Girls (%)	Mean (SD)	<30 (%)	>31 (%)	High (%)	Low (%)	
VIASANO, *Belgium*	53.4	46.6	6.6 (0.6)	20.0	80.0	42.7	57.3	178
Healthy kids in Bulgaria, *Bulgaria*	46.8	52.7	8.0 (0.8)	8.7	90.1	74.3	25.7	187
EPODE Flandre Lys, *France*	38.8	57.5	6.3 (0.6)	30.9	69.1	35.2	64.8	142
Paideiatrofi, *Greece*	46.5	45.9	7.4 (0.7)	3.2	94.4	52.8	47.2	142
MAIA, *Portugal*	51.0	48.5	7.0 (0.7)	12.4	87.1	46.0	54.0	237
SETS, *Romania*	56.8	43.2	7.4 (0.5)	17.7	82.3	53.8	46.2	173
JOGG Zwolle, *The Netherlands*	47.3	52.7	7.8 (1.0)	6.5	90.7	61.3	38.7	124
**Total**	49.8	49.2	7.16 (0.9)	14.6	84.4	52.7	47.3	1183

Notes: **^a^** The analysis includes the age of the mother only when the mother was the respondent; the age of the second parent was not assessed; Response categories: 1 = Below 20, 2 = 21–24, 3 = 25–30, 4 = 31–35, 5 = 36–40, 7 = Above 40. Number of subjects included in “age of mother” per country were: Belgium = 150, Bulgaria = 171, France = 136, Greece = 128, Portugal = 208, Romania = 147, The Netherlands = 107, Total = 1038; **^b^** Total number of subjects that provided information for the “educational level of the mother” and were included in the analysis.

**Table 2:** Rounded median values and quartiles (q_1_–q_3_) for weekly beverage intake per country.

Programme, *Country*	Water Frequency ^1^	Fruit Juices Frequency ^2^	Soft Drinks Frequency ^2^
VIASANO, *Belgium*	4 (4–5)	6 (4–6)	4 (2–5)
Healthy kids in Bulgaria, *Bulgaria*	6 (5–6)	4 (3–5)	2 (1–4)
EPODE Flandre Lys, *France*	4 (4–5)	6 (4–6)	3 (2–5)
Paideiatrofi, *Greece*	5 (5–6)	4 (4–5)	1 (1–2)
MAIA, *Portugal*	5 (4–6)	4 (2–4)	2 (1–3)
SETS, *Romania*	5 (5–6)	4 (3–5)	2 (2–4)
JOGG Zwolle, *The Netherlands*	4 (3–4)	3 (2–5)	3 (2–6)
Total	5 (4–6)	4 (3–6)	2 (1–4)

Notes: ^1^ Response categories: 1. Never 2. Less than once a day 3. Once a day 4. 2–4 times a day 5. 5–6 times a day 6. More than 6 times a day. ^2^ Response categories: 1. Never 2. Less than once a week 3. Once a week 4. 2–4 days a week 5. 5–6 days a week 6. Every day, once a day 7. Every day, more than once a day.

**Table 3:** Associations between parental practices on fruit juices and water consumption (high vs. low).

Parenting Practice	Odds Ratios (95% CI)	
	Frequency Category ^1^	OR (95% CI)
Paying attention/monitoring	Low	0.92 (0.63–0.1.35)
	Moderate	0.57 (0.38–0.86) **
	High	*Reference*
Parental allowance (If child asks for fruit juices, the parent will allow)	Low	1.33 (0.86–2.07)
	Moderate	1.20 (0.88–1.64)
	High	*Reference*
Parental allowance (Child allowed to have fruit juices whenever (s)he wants)	Low	1.28 (0.92–1.78)
	Moderate	1.14 (0.82–1.58)
	High	*Reference*
Negotiate	Low	0.92 (0.68–1.23)
	Moderate	0.95 (0.66–1.36)
	High	*Reference*
Communicate health belief (Telling the child that fruit juices are not good for him/her)	Low	1.01 (0.72–1.41)
	Moderate	0.73 (0.50–1.07)
	High	*Reference*
Communicate health belief (Telling the child that fruit juices make her/him fat)	Low	0.80 (0.56–1.15)
	Moderate	0.63 (0.41–0.98) *
	High	*Reference*
Avoid negative modelling	Low	1.01 (0.72–1.44)
	Moderate	0.87 (0.55–1.39)
	High	*Reference*
(lack of) Parental efficacy	Low	1.83 (1.04–3.20) *
	Moderate	1.84 (0.92–3.53)
	High	*Reference*
Rewarding	Low	1.36 (0.61–3.03)
	Moderate	1.71 (0.67–4.38)
	High	*Reference*
Parents drinking fruit juices together with the child ^2^	Low	0.93 (0.66–1.30)
	Moderate	1.11 (0.73–1.69)
	High	*Reference*
Home availability	Low	1.08 (0.72–1.61)
	Moderate	0.93 (0.67–1.30)
	High	*Reference*

Notes: Binary logistic regression. All associations are adjusted for country level. ^1^ Categories included in the frequencies: Low = (0) never–(1) not often, Moderate = (2) sometimes, High = (3) often–(4) always. ^2^ Categories included in the frequencies: Low = (1) never–(3) once a week, Moderate = (4) 2–4 times a week, High = (5) 5–6 times a week–(7) every day, more than once a day. *^,^ **: significance at the level of 0.05 and 0.01 respectively.

**Table 4:** Associations between parental practices on soft drinks and water consumption (high vs. low).

Parenting Practice	Odds Ratios (95% CI)	
	Frequency ^1^	OR (95% CI)
Paying attention/monitoring	Low	1.0 (0.62–1.63)
	Moderate	0.42 (0.22–0.79) **
	High	*Reference*
Parental allowance (If child asks for soft drinks, parent will allow)	Low	3.22 (2.09–4.95) ***
	Moderate	2.56 (1.64–3.99) ***
	High	*Reference*
Parental allowance (Child allowed to have soft drinks whenever (s)he wants)	Low	2.21 (1.39–3.50) **
	Moderate	1.74 (0.99–3.09)
	High	*Reference*
Communicate health belief (Telling the child that soft drinks are not good for him/her)	Low	0.58 (0.37–0.92) *
	Moderate	0.85 (0.59–1.24)
	High	*Reference*
Communicate health belief (Telling the child that soft drinks make her/him fat)	Low	0.67 (0.50–0.90) **
	Moderate	0.82 (0.56–1.20)
	High	*Reference*
Avoid negative modelling	Low	0.87 (0.63–1.20)
	Moderate	0.88 (0.60–1.29)
	High	*Reference*
(lack of) Parental efficacy	Low	2.44 (1.26–4.73) **
	Moderate	1.42 (0.66–3.06)
	High	*Reference*
Rewarding	Low	0.85 (0.18–4.01)
	Moderate	0.57 (0.10–3.17)
	High	*Reference*
Parents drinking soft drinks together with the child ^2^	Low	1.37 (0.91–2.05)
	Moderate	0.55 (0.32–0.96) *
	High	*Reference*
Home availability	Low	2.23 (1.58–3.13) ***
	Moderate	1.56 (1.05–2.23) *
	High	*Reference*

Notes: Binary logistic regression. All associations are adjusted for country level. ^1^ Categories included in the frequencies: Low = (0) never–(1) not often, Moderate = (2) sometimes, High = (3) often–(4) always; ^2^ Categories included in the frequencies: Low = (1) never–(3) once a week, Moderate = (4) 2–4 times a week, High = (5) 5–6 times a week–(7) every day, more than once a day; *^,^ **^,^ ***: significance at the level of 0.05, 0.01 and 0.001 respectively.

## Appendix A

**Table A1:** Parenting practices on sugary beverages consumption assessed in the EPHE questionnaire.

Parental Practice	Questionnaire Item
Monitoring	I pay attention to the amount of fruit juice that my child drinks
Allowing consumption	1. If my child asks for fruit juice, I will give it to him/her
	2. My child is allowed to take fruit juice whenever (s)he wants
Negotiating	I negotiate with my child on how much fruit juice/soft drinks (s)he is allowed to drink
Communicate health belief	1. How often do you tell your child that fruit juices are not good for him/her?
	2. How often do you tell your child that fruit juices make him/her fat?
Avoid negative modelling	If I would like to drink fruit juice I would restrain myself because of the presence of my child
Parental efficacy to manage child’s intake	If I prohibit my child from drinking fruit juice, I find it difficult to stick to my rule(s) if (s)he starts negotiating
Rewarding/comforting practice	I give fruit juices to my child as a reward or to comfort him/her
Drink fruit juices together with the child	How often do you or your spouse drink fruit juices together with your child?
Home availability	There are fruit juices available at home for my child
